# Flow Diverter Devices in the Treatment of Anterior Communicating Artery Region Aneurysms: Would the Regional Anatomy and the Aneurysm Location Affect the Outcomes?

**DOI:** 10.3390/brainsci12111524

**Published:** 2022-11-10

**Authors:** Mariangela Piano, Pietro Trombatore, Emilio Lozupone, Guglielmo Pero, Amedeo Cervo, Antonio Macera, Luca Quilici, Simone Peschillo, Luca Valvassori, Edoardo Boccardi

**Affiliations:** 1 Department of Neuroradiology, Niguarda Hospital, 20162 Milano, Italy; 2 Department of Neuroradiology, Ospedale San Gerardo, 20900 Monza, Italy; 3 Department of Neuroradiology, Vito Fazzi Hospital, 73100 Lecce, Italy; 4 Department of Neuroradiology, ASST Ospedale Papa Giovanni XXIII, 24127 Bergamo, Italy; 5 Department of Neurosurgey, UniCamillus-Saint Camillus International University of Health Sciences, 00131 Rome, Italy; 6 Endovascular Neurosurgery, Pia Fondazione Cardinale Giovanni Panico Hospital, 73039 Lecce, Italy; 7 Department of Neuroradiology, ASST Santi Paolo e Carlo, 20142 Milano, Italy

**Keywords:** brain aneurysm, flow diverter, endovascular treatment, anterior communicating artery

## Abstract

Background: In this study, the authors evaluated the efficacy and the safety of flow diverter devices (FDD) in anterior communicating artery (ACoA) region aneurysms, focusing on anatomical factors that could affect the outcome, such as the location of the aneurysm along the ACoA (centered on ACoA or decentered on the A1–A2 junction) and the anatomy of the ACoA region. Methods: Clinical, procedural and follow-up data were analyzed. Aneurysms were classified according to the location along the ACoA (centered or decentered on the A1–A2 junction) and on the basis of the anatomical configuration of the ACoA region. Safety was assessed by recording intraprocedural, periprocedural and delayed complications to determine the morbidity and mortality rates. The functional outcome was evaluated with the modified Rankin scale (mRS) prior to and after the endovascular procedure. To assess the efficacy, midterm and long-term clinical, angiographic and cross-sectional imaging follow-ups were recorded. Subgroup analysis according to the different ACoA regional anatomical configurations and the ACoA aneurysm locations were performed. Results: 33 patients (17 males; 16 females) with ACoA region aneurysms were treated with FDDs. 27 aneurysms were located at the A1–A2 junction (82%) while the remaining six aneurysms were centered on the ACoA. No mortality was recorded. The overall morbidity rate was 6% (2/33 procedures). Major complications occurred in 33% (2/6) of ACoA aneurysms and in the 0% of A1–A2 junction aneurysms. Mid-term and long-term neuroimaging follow-ups showed the occlusion of the aneurysm in 28/33 cases (85%). Complete occlusion rates were 93% in the A1–A2 junction aneurysms and 50% in ACoA aneurysms. Conclusions: The FDD is a safe and effective tool that can be used in the treatment of selected cases of ACoA region aneurysms. The location of the aneurysm along the ACoA and the regional anatomy of the ACoA complex could affect the efficacy and safety.

## 1. Introduction

Aneurysms of ACoA region are among the most frequent cerebral aneurysms, accounting for 23–40% of intracranial aneurysms and 12–15% of unruptured aneurysms [1,2]. Therefore, they are more likely to rupture than other types of intracranial aneurysms due to their anatomical and hemodynamic characteristics.

Although surgical treatment is a valid choice, endovascular treatment is currently the gold standard in most cases [3,4].

Treatment options for unruptured ACoA region aneurysms are multiple: coiling, balloon-assisted coiling, stent-assisted coiling, intrasaccular flow disruption devices, or FDDs. To date, there is no randomized study that demonstrates the superiority of one type of treatment over the others. 

Since their appearance in 2006, FDDs have created a revolution in the management of cerebral aneurysms [5,6,7,8]. The FDD slows the flow inside the aneurysm sac, consequently leading to intrasaccular thrombosis and aneurysmal shrinkage [9].

In recent years, the recommended application of FDDs has been extended in terms of location. FDDs have been proven to be feasible in proximal anterior and posterior circulation aneurysms, while there is still debate regarding their recommendation for distal circulation aneurysms [10,11].

There is little evidence in the literature regarding the use of FDDs for the treatment of ACoA region aneurysms. Most of the studies include A1 and distal aneurysms in their series [12,13,14].

Furthermore, recent studies highlighted the role of anatomical factors in either increasing the risk of aneurysm rupture or favoring their healing after endovascular treatment [15,16].

Our aim was to assess the efficacy and the safety of FDDs in ACoA region aneurysms, evaluating the anatomic factors that can affect the outcomes. Specifically, we focused on the location of the aneurysm neck along the ACoA and on the anatomy of the ACoA region.

## 2. Materials and Methods

### 2.1. Ethical Approval

All procedures performed were in accordance with the ethical standards of the institutional and/or national research committee and with the 1964 Helsinki Declaration and its later amendments or comparable ethical standards. Patient written informed consent was acquired before each procedure.

### 2.2. Study Design

A single-center, retrospective analysis of prospectively collected data of all endovascular treatments of ACoA region aneurysms treated via the implantation of FDDs was conducted.

From December 2009 to December 2020, 33 consecutive patients (17 males, 16 females; 10–75 years old with an average age of 53) with unruptured ACoA region aneurysms were treated with FDDs. A total of 34 procedures were performed on 33 patients.

Inclusion criteria were:

- Cerebral aneurysm of the ACoA treated via implantation of FDD;
- Availability of clinical and procedural data;
- Availability of at least one angiographical FUP and clinical data at 3 months.

Exclusion criteria were:

- The localization of the aneurysm on the A1 or A2 segments of the anterior cerebral artery;
- Endovascular without FDDs.

All the measurements (maximum diameter of the sac, maximum diameter of the neck) of the aneurysms were performed on 3D acquisitions directly on the reconstruction console of the angiographic system.

Clinical, procedural and follow-up data were analyzed in order to evaluate the safety and effectiveness of the treatment with FDDs in ACoA aneurysms. Aneurysms were classified according to the location of the neck along the ACoA:

- ACoA aneurysm: for those aneurysms located in the center of ACoA;
- A1–A2 junction aneurysms: for those located at the A1–A2 junction.

Regional anatomy of the ACoA was classified based on the diameters of both A1 segments [17]:

- H1: both A1 segments have the same diameter;
- H2: A1 segments have different diameters, with <50% difference between the sides;
- H3: A1 segments have different diameters, with ≥50% difference between the sides;
- Y type, no A1 segment on one side.

Safety was analyzed recording intraprocedural, periprocedural (0–30 days after procedure) and delayed complication (from 30 days after the procedure) in order to determine the morbidity and mortality rates. Morbidity was assessed with every severe adverse event (SAE, events with clinical relevance and permanent) and mild adverse events (MAE, events with clinical relevance and transient). Asymptomatic adverse events (AAE, events without any clinical relevance) and the technical notes (TN) were also recorded.

The mRS was used to evaluate the disability degree of each patient before and after the endovascular treatment (6 months follow-up).

Imaging follow-up involved CT/MRI follow-up at 1 month, DSA follow-up at 6 (with or without CT/MRI), and CT/MRI follow-up at 12/24 months.

Efficacy was assessed by applying the angiographic classification scheme for grading the aneurysm occlusion Raymond–Roy occlusion classification (RROC): complete occlusion (A), neck remnant (B) and aneurysm remnant (C) [18].

In addition, we also analyzed the aneurysmal sac modification (shrunk, unchanged, increased).

### 2.3. Statistical Analysis

Descriptive statistical analysis was conducted. Data are presented as mean, standard deviation and range for continuous variables, and as frequency and percentage for categorical variables.

## 3. Results

### 3.1. Patient Population (Table 1)

A total of 34 procedures were performed on 33 patients with unruptured ACoA region aneurysms. Of the 33 patients, 26 were asymptomatic (79%); 3 presented for visual impairment, 3 for headache, 1 for lower limb paresis.

The disability degree of each patient was assessed before the endovascular treatment using the modified Rankin scale (mRS): mRS score was 0 in 28/33 patients (85%), 1 in 4/33 patients (12%) and 4 in 1/33 patient (3%).

### 3.2. Aneurysms Characteristics (Table 2)

All the aneurysms treated were unruptured at the time of the procedure.

**Figure 1:**
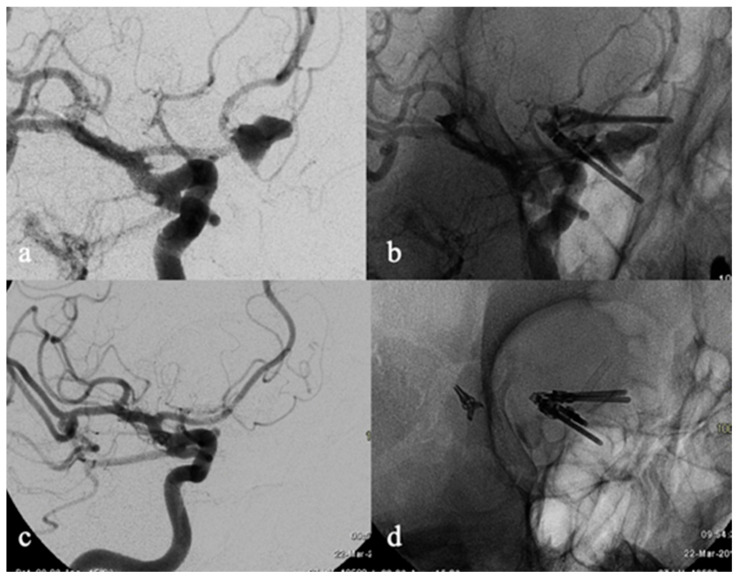
Fifty-year-old female with a recurrence of right A1–A2 junction aneurysm clipped 3 months before in emergency because of large intraparenchymal hematoma and SAH. Subtracted and unsubtracted angiogram (**a**,**b**) show the recurrence of the aneurysmal sac that was treated via implantation of a FDD along the right A1–A2 segments. Six-month DSA follow-up (**c**,**d**) demonstrated the exclusion of the aneurysm (Class A RROC).

Eleven (33%) aneurysms were small (<10 mm in maximum diameter), 19 (58%) were large (≥10 and <25 mm), and 3 (9%) were giant (≥25 mm), with a mean size of 13.9 mm (median: 10 mm; range: 1–45 mm). Thirty aneurysms were saccular (91%), three dissecting (9%). Fifteen (45%) aneurysms were recurrent (Class C RROC) (Figure 1): 13 aneurysms were ruptured (11 treated with coiling, 1 with clipping, and 1 with both coiling and clipping) and 2 aneurysms were unruptured (both treated with coiling).

Twenty-seven aneurysms were A1–A2 junction aneurysms (82%), six aneurysms were ACoA aneurysms (18%).

Based on the abovementioned anatomical classification of the ACoA region, 18/33 ACoA belong to H1 type (55%), 10 to H2 type (30%), 3 to H3 type (9%) and 2 to Y type (6%).

In the subgroup A1–A2 junction aneurysms, 15 were H1 type, 8 were H2 type, 3 were H3 type and one was Y type. In the subgroup ACoA aneurysms, 3 were H1 type, 2 were H2 type and one was Y type.

### 3.3. Endovascular Procedure

All procedures were performed under general anesthesia on a biplane angiographic system (Philips Integris Allura Unit 12 and Philips Azurion 7 B20/15 with Clarity IQ; Philips Medical Systems, Best, The Netherlands). A biaxial system consisting of a 6 French guiding catheter and a microcatheter were used via the right femoral approach in 32/34 procedures (94%). An intermediate catheter was used only in 2/34 procedures (6%).

In all cases, post-treatment DSA at 10 and 30 min after FDD deployment was routinely performed; a CT scan was also performed in all cases to immediately rule out hemorrhagic complications.

All the procedures were in elective settings. Mean procedure time was 95 min (median, 90 min; range, 35–165 min). FDD placement was technically successful in all cases.

**Figure 2:**
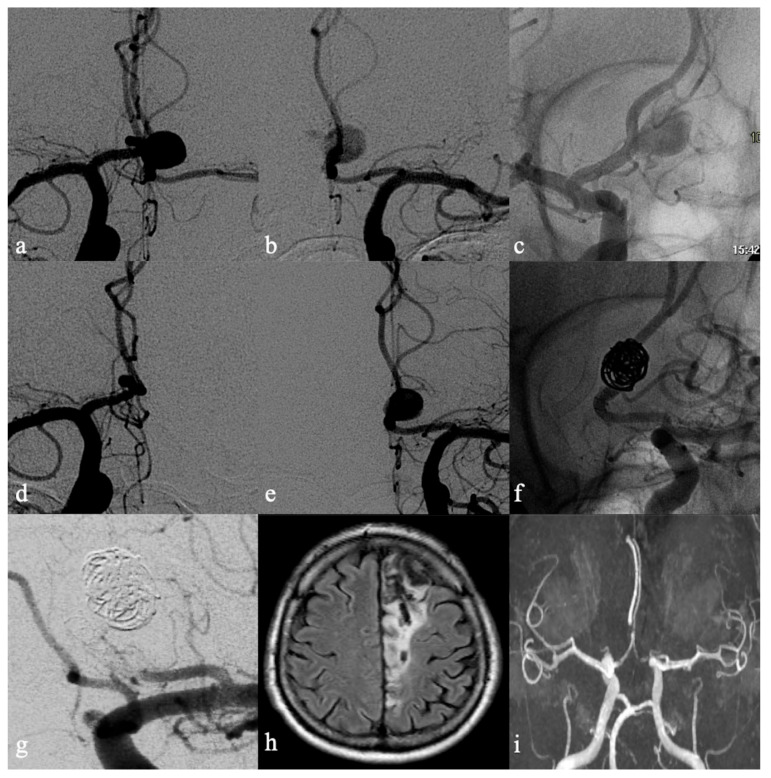
Fifty-year-old male with incidental finding of 10 mm ACoA aneurysm. Frontal angiograms (**a**,**b**) highlight the flow competition between the ACAs which allowed the opacification of the aneurysm during selective injection of both ICA with a slight prevalence from the right side. The aneurysm was treated via implantation of FDD along the right A1–A2 segments as shown in the unsubtracted angiograms (**c**). Six-month DSA FUP shows the exclusion of the aneurysm from the right anterior circulation (**d**) with persistency of the aneurysm which is injected by the left anterior circulation (**e**). A second FDD along the left A1-A2 segments with adjunctive coiling was performed (**f**). After 2 h, patient experienced acute right hemiplegia due to intrastent thrombosis and occlusion of left ACA (**g**); any attempts to recanalize the ACA were ineffective. Twelve months MR FUP demonstrated the chronic transformation of the infarct in the left ACA territory (**h**) and exclusion of the aneurysm (Class A RROC) in MR-angiography (**i**).

A total of 34 procedures were performed. FDD was deployed from the ipsilateral A2 to the ipsilateral A1 (ipsilateral deployment) in 29 procedures (85%) and from the contralateral A2 to the ipsilateral A1 (cross deployment) in 4 procedures (12%). One patient with an ACoA aneurysm underwent two treatments due to inefficacy of the first one: at first, the FDD was deployed from the right A2 to the ipsilateral A1; 21 months later, a second FDD was deployed from the left A2 to the ipsilateral A1 (H configuration, 3%) because the total residual filling of the aneurysm from the left A1 (Figure 2).

In the subgroup of aneurysms treated with ipsilateral deployment, 18/29 belonged to H1 anatomical type (62%), 9/29 to H2 type (31%) and 2/29 to H3 type (7%); none had a type Y anatomy.

In the subgroup of aneurysms treated with cross deployment, 2/4 belonged to Y anatomical type (50%), 1/4 to H2 type (25%) and 1/4 to H3 type (25%); none had a type H1 anatomy.

Analyzing the subgroup of the ACoA aneurysms, 4/6 aneurysms were treated with ipsilateral deployment (67%), 2/6 aneurysms were treated with cross stenting (33%). 

Analyzing the subgroup of the A1–A2 junction aneurysms, 25/27 aneurysms were treated with ipsilateral deployment (67%), 2/27 aneurysms were treated with cross stenting (33%).

A pipeline embolization device (PED, Medtronic, Irvine, CA, USA) was used in 22 treatments (65%); a flow-redirection endoluminal device (FRED, MicroVention, Aliso Viejo, CA, USA) was used in 6 treatments (18%); Silk Vista (Balt, Montmorency, France) was used in 5 treatments (15%); p64 A flow modulation device (Phenox, Bochum, Germany) was used in 1 treatment (2%).

Additional coiling was performed in six procedures (18%) to reduce the risk of aneurysmal rupture due to acute thrombosis; among these, a jailing technique was performed in three cases (50%).

### 3.4. Pharmacological Management

All patients were pharmacologically prepared with dual antiplatelet therapy (acetylsalicylic acid (ASA) 100 mg/day and ticlopidine 250 mg twice a day) 7 days before the treatment. Dual antiplatelet therapy was continued for 1 month after the treatment, followed by 3 months of single antiplatelet therapy with ASA, except for five cases in which the dual antiplatelet therapy was continued for 2 months after the procedure while ASA was discontinued after 6 months. 

### 3.5. Safety (Table 3)

Overall mortality and morbidity rates were 0% and 6% (2/33), respectively.

Asymptomatic adverse events (with no clinical relevance) occurred in 18% of cases (6/33). All complications were ischemic/thromboembolic events. No hemorrhagic complications (aneurysm rupture or intraparenchymal hemorrhage) were reported.

#### 3.5.1. Morbidity

Overall morbidity was 6% (2/33): two SAE (one in the intra/periprocedural period, one in the delayed period) and zero MAE.

One patient treated with H configuration suffered from aphasia and right hemiplegia a few hours after the treatment due to thrombosis of the FDD placed in the left ACA and consequent ischemic lesions (mRS shifted from 0 to 4) (Figure 2). One patient (aneurysm treated with cross deployment of the FDD) experienced the onset of behavioral frontal lobe syndrome a few months after the treatment. MRI showed multiple ischemic lesions in the territory of the ACA covered by the FDD (mRS shifted from 0 to 1).

Morbidity was 33% in the ACoA aneurysms (2/6), and 0% in A1–A2 junction aneurysms (0/27). The abovementioned SAEs occurred in one case of H1 type and in one case of Y type configurations. Morbidity was 6% in H1 type (1/18), 0% in H2 and H3 type (0/10 and 0/3, respectively) and 50% in Y type (1/2). Morbidity for ipsilateral deployment was 0% (0/29), for cross deployment was 25% (1/4) and for H deployment was 100% (1/1).

#### 3.5.2. Asymptomatic Adverse Events (AAE)

Six asymptomatic adverse events (AAE) were recorded (18%, 6/33), all intraprocedurally. Five cases (15%, 5/34) of acute intrastent platelet aggregation immediately after deployment of the FDD were recorded. Prompt resolution of the platelet aggregation was achieved after intravenous administration of Abixicimab in four cases; in one case Abiciximab was ineffective so deployment of a closed cell stent (Leo Baby; Balt, Montmorency, France) was needed to resolve in-stent thrombosis and to improve wall apposition of the FDD. In one case asymptomatic ischemic lesions in the territories supplied by the Heubner and frontopolar arteries were reported in the post-treatment CT scan (1/33, 3%). AAEs were 3/27 in A1–A2 junction aneurysms (11%) and 3/6 in ACoA aneurysms (50%). AAE rate was 22% in H1 type (4/18), 20% in H2 type (2/10), 0% in H3 type (0/3) and 0% in Y type (0/2). AAEs were 6/29 in ipsilateral deployment (21%), 0/4 in cross deployment (0%) and 0/1 in H deployment (0%). 

### 3.6. Efficacy (Table 4)

#### 3.6.1. Functional Outcome

Before the endovascular procedure, 28 of the 33 (85%) patients were asymptomatic (mRS = 0), 4 (12%) showed minimal disability (mRS = 1), and 1 (3%) mild/severe disability (mRS = 4). Clinical follow-up was available for all the patients. At the last clinical follow-up, 26 patients (79%) had no symptoms (mRS = 0), 2 showed severe disability (6%) (mRS = 4) and 5 (15%) showed light disability (mRS = 1); 2 patients presented a worsening of mRS due to the occurrence of severe adverse events. One shifted from 0 to 4, and another one shifted from 0 to 1.

#### 3.6.2. Imaging Follow-Up

The mean radiologic follow-up was 20 months (range: 6 months–10 years).

CT/MR angiography at 1 month from the treatment was available for 21/34 procedures (62%). All patients had a DSA at 6 months, associated with MR imaging in 16/34 procedures (47%). CT/MR angiography or DSA at 12 months was available for 16/34 procedures (47%) and at 24 months for 15/34 treatments (44%). Of 34 procedures, 3 had a MR angiography at 5 years (9%) and one case had a MR angiography at 10 years (3%).

##### Short-Term Results

CT/MR angiography at 1 month from the treatment was available for 21/34 procedures (62%): complete occlusion (Class A RROC) was reported in 10/21 cases (48%), neck remnant (Class B RROC) in 4/21 cases (19%) and aneurysm remnant (Class C RROC) in 7/21 (33%). 

##### Mid-Term Results

All patients had a DSA at 6 months, associated with MR imaging in 16/34 procedures (47%). Complete occlusion (Class A RROC) was reported in 25/34 cases (74%), neck remnant (Class B RROC) in 3/34 cases (9%), and aneurysm remnant (Class C RROC) in 6/34 (17%). Specifically, two aneurysms passed from grade B to A, three from grade C to A, and one from grade C to B.

**Figure 3:**
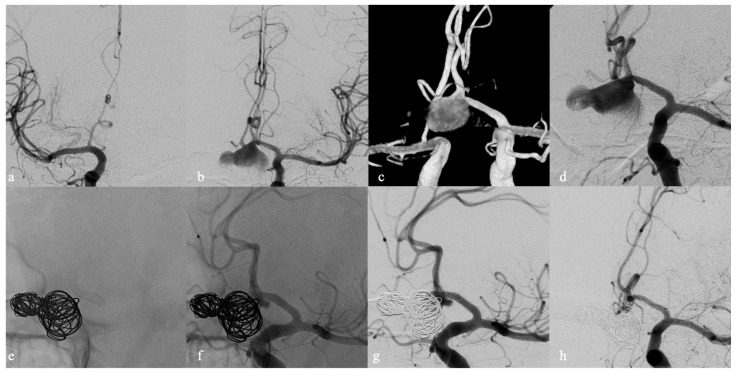
Sixty-one-year-old male with onset of visual impairment and finding of a large ACoA region aneurysm. Frontal angiograms (**a**,**b**) during selective injection of both ICA show how the aneurysm is injected only through the left ICA. Three-dimensional reconstruction (**c**) and frontal working projection (**d**) better disclosed the location of the aneurysm neck at the left A1–A2 junction. Unsubtracted (**e**,**f**) and subtracted (**g**) oblique angiograms after the implantation of FDD along left A1–A2 segments and the deposition of platinum coils in the aneurysmal sac. Six-month DSA follow-up (**h**) demonstrated the exclusion of the aneurysm (Class I RROC).

The overall rate of complete/near-complete occlusion (Class A and B RROC) was 85% (28/33). In the subgroup of A1–A2 junction aneurysms, complete/near-complete occlusion rate was 93% (25/27) (Figure 3). Twenty-five out of twenty-seven aneurysms were treated with ipsilateral deployment with a recurrence rate of 4% (1/25), while 2/27 were treated with cross deployment with a recurrence rate of 50% (1/2).

In the subgroup of ACoA aneurysms, complete/near-complete occlusion rate was 50% (3/6). Three out of six aneurysms were treated with ipsilateral deployment with a recurrence rate of 67% (2/3), 2/6 were treated with cross deployment with a recurrence rate of 50% (1/2) and 1/6 was treated with H deployment with no recurrence.

Complete/near complete occlusion rate was 94% in H1 type (17/18), 60% in H2 (6/10), 100% in H3 type (3/3) and 100% in Y type (2/2).

In the subgroup of aneurysms treated with ipsilateral deployment, the overall complete/near complete occlusion rate was 89% (25/28); specifically, the complete/near-complete occlusion rate was 94% in H1 anatomical type (16/17), 75% in H2 type (6/8) and 100% in H3 type (3/3). In the subgroup of aneurysms treated with cross deployment, the overall complete/near-complete occlusion rate was 50% (2/4); specifically, the complete/near-complete occlusion rate was 100% in Y anatomical type (2/2), and 0% in H2 type (0/2). A patient treated with H deployment, belonging to H1 anatomical type, reported a complete occlusion.

Shrinkage of the aneurysmal sac occurred in 16/34 cases (47%) in the MR imaging: specifically, the complete disappearance of the aneurysm occurs in 7 cases while a partial dimensional reduction of the sac occurs in the remaining 9 cases.

##### Long-Term Results

CT/MR angiography or DSA at 12 months was available for 16/34 procedures (47%) and at 24 months for 15/34 treatments (44%) and confirmed the results of the 6-month follow up in all cases. Three out thirty-four procedures had an MR angiography at 5 years (9%) and one case had a MR angiography at 10 years (3%) that confirmed the previous results. 

#### 3.6.3. Flow Modification

The overall rate of flow modifications of vessels after the treatment was 68% (23/34).

Flow modifications occurred in 20/29 cases (69%) treated with ipsilateral deployment. The most common modification in this subgroup was that the contralateral A2 became supplied by its ipsilateral A1 (18/29, 62%), which showed an increase in diameter in 8/29 (28%). In one case (3%), despite contralateral A1 hypertrophy, the contralateral A2 was supplied by leptomeningeal collateral circulation from the ipsilateral MCA and PCA rather than by its ipsilateral A1. In another case (3%) reported the occlusion of the intra-stent A1 with the ipsilateral A2 supplied by the contralateral A1 through the ACoA.

When the FDD was deployed from the contralateral A2 to the ipsilateral A1, 3/4 cases (75%) had a flow change in the A2 covered by the stent, which became supplied by leptomeningeal collateral circulation from the ipsilateral MCA and PCA.

## 4. Discussion

The ACoA is a short blood vessel that connects the bilateral anterior cerebral arteries. It is located above the optic chiasm and corresponds to the inferior portion of the lamina terminals [19].

It is almost unique in terms of its high variability of configuration and hemodynamics. Kirgis et al. [20] classified the ACoA into simple and complex according to the morphology of afferent blood vessels. Constitutional variations in caliber of A1 segment radically changes regional hemodynamic and flow across the ACoA. These peculiar features are co-responsible for the genesis of the aneurysm and have an important role in the growth of the aneurysmal sac as well in the recurrence after the endovascular treatment.

Treatment options for unruptured ACoA region aneurysms are multiple: coiling, remodeling, stent-assisted coiling, intrasaccular flow disruption devices, or FDDs. FDDs work on the diversion of flow across the aneurysm neck while in the others endovascular techniques the goal of the procedure is the occlusion of sac of the aneurysm. For these reasons, anatomical and hemodynamic features of the ACoA complex are key factors that can significantly affect the efficacy of the treatment with FDDs.

In our series, flow diversion for the treatment of ACoA aneurysms showed high rates of aneurysm occlusion. Complete/near-complete occlusion (Class A and B RROC) was achieved in 85% of cases at 6 months. These results are comparable with those described in the series of Colby et al. [21] and in the meta-analysis by Cagnazzo et al. [22], which reported occlusion rates of 81% and 87%, respectively.

These results are also comparable to alternative endovascular treatments. Choi et al. [23] reported similar occlusion rates (86.9%) in their series with 184 aneurysms treated with stent-assisted coiling. Similar results were reported by Fang et al. [2] on more than 1500 ACoA aneurysms treated with coiling (85%). FDD occlusion rate actually appears to be slightly higher than WEB treatment, which is around 68.8% [24].

In cases where it was available, the long-term follow up confirmed the stability of the occlusion. This data is important as it confirms that assumption that once the aneurysm is closed it doesn’t recanalize over time, unlike other types of treatments in which the aneurysm can recur even after several years.

Regarding the location of the aneurysm along the ACoA, the complete/near-complete occlusion rate was 93% versus 50% in the A1–A2 junction aneurysms and in ACoA aneurysms, respectively. This result suggests that in our experience the effectiveness of the FDD treatment is greater in case of A1–A2 junction aneurysms rather than in ACoA aneurysms. A1–A2 junction aneurysms hemodynamically belong to the anterior cerebral artery and therefore FDDs work as if they are a side-wall aneurysm. Conversely, due to the centered location of AcoA aneurysms in the middle of the artery, they are prone to be hemodynamically supported by both the ACAs; thus, the possibility of the inefficacy of the flow diversion is higher due to the flow competition between the two ACAs. Furthermore, FD deployment in ACoA aneurysms seems to have more SAE than A1–A2 junction aneurysms, with a morbidity of 33% versus 0%, respectively.

Several authors have highlighted the importance of the anatomy of the region in the healing of ACoA aneurysms. It is well known how this anatomy can be variable due to the presence of fenestration, symmetric A1s, hypoplastic A1, or aplastic A1. Choi et al. [23] suggested A1 hypoplasia as predictor of recanalization after stent-assisted coiling; the same hypothesis is supported by Tarulli et al. [25] about coiling, suggesting that the predominance of blood flow from one side may lead to mold and flatten the coil masses. 

Conversely, Cortese et al. [24] reported that ACoA aneurysms with hypoplastic/aplastic A1 treated with WEB are associated with better angiographic outcomes than those with bilateral A1.

How the anatomy of the ACoA region relates to FDD treatment is not yet fully understood. Pagiola et al. [17] tried to find a correlation in their series of 30 ACoA aneurysms treated by FDD deployment, reporting the lowest rates of occlusion in H3 types. These results were not confirmed in our study: we reported the highest complete/near complete occlusion rate in H3 type (100%) and H1 type (94.5%), while reporting the lowest in H2 type (60%). However, this last result is probably influenced by the choice of cross deployment in 2/10 cases of H2 type that led to aneurysm remnants in both cases; as confirmation of this, the complete/near complete occlusion rate increases from 60% to 75% (6/8) considering only those treated with ipsilateral deployment. 

Furthermore, in contrast to Pagiola et al. [17], who reported the highest rates of complications in patients with hypoplastic A1 (especially in the H3 type), we found the highest SAE and AAE rates in H1 type (6% and 22%, respectively).

Focusing on the Y type aneurysms (not included in the Pagiola’s case series), we reported the highest morbidity (50%) despite a good occlusion rate (100%).

Ipsilateral deployment was the most frequent choice and proved to be the most effective treatment as the healing of the aneurysm (RR grade A or B) was obtained in 89% of the cases (25/28). 

Cross-deployment reported a success rate of only 50% (2/4 cases), considerably lower than ipsilateral deployment, suggesting the superiority of the latter treatment. The hypothesis we have assumed regarding this lower occlusion rate is that the aneurysm probably continues to be filled by the flow towards the A2 covered by the stent. As confirmation of this, in the two cases in which the aneurysm healed, the A2 jailed by the stent, was retrograde filled by leptomeningeal collateral circulation from the MCA and PCA. The same findings were reported by Colby et al. [21] in the three healed aneurysms treated with cross deployment.

The most common flow modification in cases treated with ipsilateral deployment was that the contralateral A2 became supplied by its ipsilateral A1 (18/29, 62%). Focusing on the subgroups with H2 and H3 anatomical setting, 75% (6/8) and 100% (3/3) of the cases reported an increase in caliber of the contralateral A1, respectively. It is interesting to underline that in one of these three H3 type cases, contralateral A2 was supplied by leptomeningeal collateral circulation from ipsilateral MCA and PCA probably because the flow through the hypertrophied A1 was not enough to support the demands of the cerebral tissue. None of these angiographic changes in blood flow resulted in clinical symptoms.

Fifteen patients had previously been treated with other techniques (coiling and/or clipping) and were retreated because an aneurysmal remnant was detected. Among these, two patients admitted with subarachnoid hemorrhage underwent a loose coiling in emergency to avoid the imminent risk of rebleeding and then the treatment was completed by deploying a FDD within one month, once the risk of SAH-induced vasospasm has passed. This strategy can be very useful in wide-neck aneurysms where a particularly dense coiling could lead to the occlusion of the afferent vessels or in dissecting aneurysms, in which it is necessary to reconstruct the vessel wall [26].

Overall morbidity was 6% (2/34): two SAEs (one in the intra/periprocedural period, and one in the delayed period) and zero MAE, comparable to that reported by Pagiola et al. [17], Colby et al. [21], and Cagnazzo et al. [22], which were 3.3%, 9% and 8.6%, respectively. Furthermore, morbidity in our series is comparable to that reported by other techniques such as stent assisted coiling [27] and WEB [28].

Acute in-stent thrombosis was the most common intraprocedural adverse event and occurred in 6/34 procedures (18%). Management of the thromboembolic complications could be challenging. The general consensus is to use intravenous antiplatelet agents, currently considered the first choice because platelet aggregation represents a primary component of acutely formed arterial thrombi, [29] and to avoid fibrinolytic agents because associated with greater morbidity and mortality than rescue therapy with glycoprotein IIb/IIIa inhibitors. [30] In the reported cases we used abciximab [31], which worked in 67% of cases (4/6). Regarding the remaining two cases, in one case the occlusion was resolved by angioplasty with a balloon catheter (Scepter, Microvention, Aliso Viejo, CA, USA) followed by the placement of a closed-cell stent, without clinical consequences; in the other case we were unable to resolve the occlusion despite an attempt at intrastent thrombectomy and of the FDD’s removal, resulting in ischemia of the territory supplied by the anterior cerebral artery (mRS = 4).

### Limitations

The limitations of this study are its retrospective design, the non-randomized nature and the small number of patients. Other limitations consist in the lack of balance among the FDD deployment technique, the location of the aneurysm (ACoA vs. A1-A2 junction), and the anatomical configurations of the ACoA region. Large randomized prospective clinical studies are needed to define how anatomical factors can affect the efficacy and the safety in the treatment of the aneurysms of the anterior communicating artery region.

## 5. Conclusions

Flow diversion in ACoA region aneurysms seems to be safe and effective, with high rates of aneurysm healing in the mid- and long-term follow-ups and acceptable morbidity rates.

The location of the aneurysm along the ACoA and the anatomical configuration of the artery are key factors that affect the efficacy and safety of the treatment with FDDs and therefore should be carefully evaluated before endovascular procedure.

## Figures and Tables

**Table 1:** Patients Demographics and Clinic.

Patients	33
Sex (male)	17 (52%)
Age (mean) [range]	53 [10–75]
Symptoms	
Asymptomatic	26 (79%)
Symptomatic	7 (21%)
Visual impairment	3 (9%)
Headache	3 (9%)
Paresis	1 (3%)

**Table 2:** Aneurysm Characteristics.

N. Aneurysm	33
Ruptured	13 (39%)
Unruptured	20 (61%)
Location	
A1–A2 junction	27 (82%)
ACoA	6 (18%)
ACoA anatomy region	
H1	18 (55%)
H2	10 (30%)
H3	3 (9%)
Y	2 (6%)
Morphology	
Saccular	30 (91%)
Dissecting	3 (9%)
Dimension	
Small (<10 mm)	11 (33%)
Large (≥10 and <25 mm)	19 (58%)
Giant (≥25 mm)	3 (9%)
Status	
First treatment	18 (55%)
Retreatment after coiling	13 (39%)
Retreatment after clipping	1 (3%)
Retreatment after coiling + clipping	1 (3%)

**Table 3:** Safety.

	Mortality	Morbidity	AAE
Overall	0	2/33 (6%)	6/33 (18%)
Location			
A1–A2 junction	0	0	3/27 (11%)
ACoA	0	2/6 (33%)	3/6 (50%)
ACoA anatomy region			
H1	0	1/18 (6%)	4/18 (22%)
H2	0	0	2/10 (20%)
H3	0	0	0
Y	0	1/2 (50%)	0

SAE: severe adverse event; MAE: mild adverse event; AAE: asymptomatic adverse event.

**Table 4:** Efficacy.

	RROC A	RROC B	RROC C
Overall	25/33 (76%)	3/33 (9%)	5/33 (15%)
Location			
A1-A2 junction	22/27 (82%)	3/27 (11%)	2/27 (7%)
ACoA	3/6 (50%)	0	3/6 (50%)
ACoA anatomy region			
H1	16/18 (89%)	1/18 (5.5%)	1/18 (5.5%)
H2	5/10 (50%)	1/10 (10%)	4/10 (40%)
H3	2/3 (67%)	1/3 (33%)	0
Y	2/2 (100%)	0	0

RROC: Raymond-–Roy occlusion classification.

## Data Availability

The data presented in this study are available on request from the corresponding author.
